# Niemann-Pick Disease on Bone Marrow Trephine: A Rare Manifestation

**DOI:** 10.7759/cureus.19246

**Published:** 2021-11-04

**Authors:** Hira Qadir, Mahad M Baig, Anas Adil, Maria Aisha, Izzan Raees

**Affiliations:** 1 Pathology, Dow International Medical College, Dow University of Health Sciences, Karachi, PAK

**Keywords:** niemann-pick disease, bone trephine, foam cells, sphingomyelinase, hepatosplenomegaly

## Abstract

Niemann-Pick disease has an autosomal recessive inheritance pattern and occurs due to a deficiency of a lysosomal enzyme, sphingomyelinase. It causes variable clinical signs and symptoms such as hepatosplenomegaly, delayed milestones, and peripheral cytopenia due to bone marrow involvement. Here, we report a case of a child who presented with hepatosplenomegaly and pancytopenia, who was later found to have Niemann-Pick disease on bone marrow examination. This case highlights the case presentations of this rare disease and the importance of bone marrow trephine in prompt diagnosis and management of a patient.

## Introduction

Niemann-Pick disease (NPD) is an autosomal recessive disease that manifests with defective lysosomes that accumulate macromolecules; hence, it is described as a lysosomal storage disorder [1-3]. The most notable of the macromolecules to accumulate are lipids such as sphingomyelin and cholesterol, with inclusions being found commonly in the various viscera such as the spleen, liver, and bone marrow [1,4,5]. The result is often hepatosplenomegaly and the distinctive cherry-red spots on the retina [5]. Very little is known about this disease, partly due to its rarity, with one study estimating the incidence to occur between one in 150,000 and one in 250,000 people [6]. 

In Pakistan, despite consanguineous marriages being relatively common in this country, even less has been reported on this rare disease, with the first genetic demographic study having been completed in March 2019 by Huma Arshad Cheema and her colleagues in Lahore [7]. Although our team was unable to find a comprehensive study of autosomal recessive disorders in Pakistan, one study has concluded that a higher rate of morbidity and mortality exists in the UK among Pakistani children due to autosomal recessive conditions with association to the custom of consanguineous marriages [8]. Therefore, this disease is more prevalent in the West, especially type A Niemann-Pick disease which is exhibited at higher rates among Ashkenazi Jews [9].

Various reviews and reports consequently appear to be in consensus that further research is needed from which an appropriate conclusion can be drawn for potential therapies and diagnostic methods [10,11]. This case report shall seek to highlight the case presentations of this rare disease so that the issue of under-diagnosis can be more readily addressed [11]. This elaboration of data shall contribute and encourage the discovery of clinical findings for future investigations as efforts are made to better understand this disease.

## Case presentation

A two-year-old male child was born of consanguineous marriage with normal delivery. During the historical examination of the patient's parents, no incidence of Niemann-Pick disease was discovered in the family. The patient's only sibling is his older brother, who is free of disease. The parents complained of gradually increasing generalized abdominal distention, which was not associated with feeding and bowel habits in the patient. They also revealed that the patient had a history of low-grade intermittent fever. The mother reported feeding difficulties and excessive crying of the child. Developmental history revealed delayed milestones in the form of neck-holding achieved at 10 months. No other delayed milestones were reported. Intrauterine infections and family history for any similar illness were non-significant. On general examination, the child was malnourished, irritable, and afebrile. All vitals were within normal range. On abdominal examination, hepatosplenomegaly was found. Fundus examination depicted a mildly pale disc and bilateral cherry-red spots. Neurological examination was non-significant for dystonia, ataxia, and seizures. Complete blood counts showed hemoglobin 9.0 g/dL, hematocrit (HCT) 30%, mean corpuscular volume (MCV) 90 fL, mean corpuscular hemoglobin (MCH) 27 pg, white blood cells 2.0 x 10^9/L, and platelets 100 x 10^9/L (Table 1). Liver function tests were within normal reference value. Ultrasound of the abdomen revealed gross hepatosplenomegaly with spleen size reaching up to 19 cm and mildly enlarged liver. Electroencephalography and MRI brain were normal. Additionally, an electrophoresis study was completed, which yielded no significant findings.

**Table 1:** Tabulated Entries of the Complete Blood Count (CBC)

Item	Value
Hemoglobin (Hb)	9.0 g/dL
Hematocrit (HCT)	30%
Mean Cell Volume (MCV)	90 fL
Mean Corpuscular Hemoglobin (MCH)	27 pg
White Blood Cell Count (WBC)	2.0 x 10^9 ^/L
Platelets (PLT)	100 x 10^9^ /L

**Figure 1:**
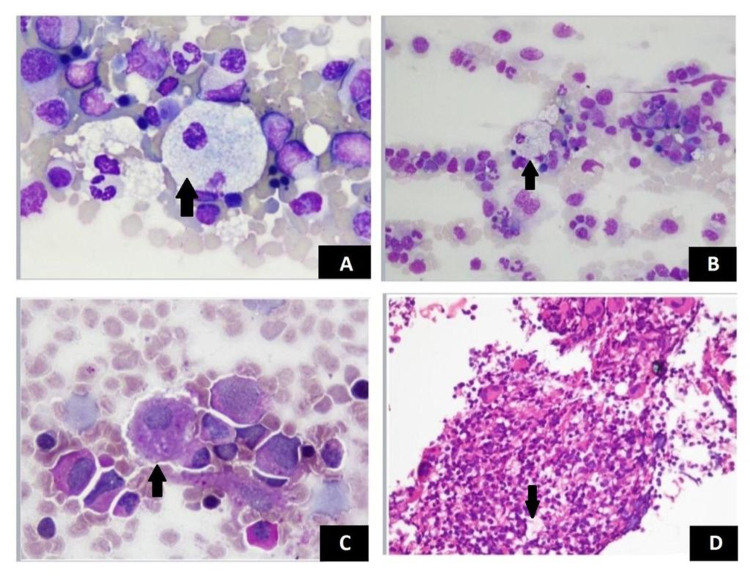
Foam Cells: Bone Marrow Aspirate with Leishman Stain (A, B) and PAS Stain (C) and Bone Trephine H&E Section (D) PAS = Periodic acid-Schiff stain H&E = Haemotoxylin and eosin stain

Since no evident cause was recognized thus far, the primary physician requested a bone marrow biopsy for the workup of pancytopenia. Bone marrow aspirate showed trilineage hematopoiesis with abnormally enlarged lipid-filled macrophages (foam cells). Foam cells have a soap-bubble appearing cytoplasm with eccentric nuclei (as shown in Figures 1A, 1B). These cells show positive staining with periodic acid-Schiff stain (Figure 1C). Bone trephine yielded a cellular specimen showing similar abnormal macrophages (Figure 1D). Clinical details and bone marrow morphological findings are therefore suggestive of a lysosomal storage disorder. The primary physician was promptly informed about the bone marrow findings. Subsequent workup of enzyme assays showed sphingomyelinase deficiency. Based on these clinical and laboratory findings, the diagnosis of Niemann-Pick disease was made. The patient received symptomatic treatment and was discharged with appropriate disease prognosis counseling. The patient is awaited for follow-up.

## Discussion

Niemann-Pick disease has an autosomal recessive inheritance pattern. It is mainly of three types: A, B, and C. Type A and B occur due to deficiency of the lysosomal enzyme sphingomyelinase. In contrast, type C occurs due to a deficient lysosomal membrane protein which is responsible for the in-and-out movement of cholesterol through the cell. Niemann-Pick disease presents with a variety of clinical signs and symptoms, which may include variable hepatosplenomegaly, fever, dysphagia, dystonia, ataxia, cherry-red spots in the eye, developmental delay, etc. Variation in the signs and symptoms of different age groups has been observed, including that of clinical presentation and progression of the disease. The disease tends to be less severe and has a slower progression when there is a delay in the onset of neurological symptoms and vice-versa [1]. Many studies have estimated the incidence of Niemann-Pick disease worldwide to be 0.5 to one per 100,000 [3]. Consanguineous marriage is a major contributing factor in the development of this rare autosomal recessive disorder, especially in our part of the world, which highlights its importance [2]. Niemann-Pick disease type A and type B refer to the classic infantile form and visceral juvenile form of the disease, respectively.

Niemann-Pick Disease type A usually begins within the first few months of life, often sparing the neonatal period, unlike type B, which may manifest at any time in life. As far as type A is concerned, failure to thrive is usually the presenting feature, whereas hepatosplenomegaly is usually found on abdominal examination of such infants, which helps in the diagnosis of the disease [4]. Although development usually progresses at a normal pace within the first six months of life, it somewhat quiesces onwards, contributing to the CNS manifestations of the disease in type A including psychomotor retardation, decreased spontaneous movements, dysphagia, and worsening hypotonia. Other symptoms include feeding difficulties and loss of reflexes. In contrast, type B has no neurological involvement and is characterized by progressive splenomegaly and peripheral cytopenia accompanied by variable signs of liver failure. Other symptoms include feeding difficulties and loss of reflexes. Most infants with Niemann-Pick disease type A lack the ability to sit without support [5]. Ophthalmologic examination reveals cherry-red spots in the macula, which causes a decrease in central vision in about half of patients [5]. Bone marrow involvement by macrophages may occur, resulting in peripheral cytopenia. Pulmonary complications arise due to the accumulation of sphingomyelin-containing macrophages in the lungs, which ultimately results in interstitial lung disease; this, together with recurrent pulmonary infections, leads to respiratory failure, making the prognosis of type A disease worse. Death usually occurs by the age of three years. Niemann-Pick disease type B is less severe and bears a variable prognosis. Niemann-Pick disease type C can occur in infancy, childhood, and adulthood. It frequently involves the brain, bone marrow, liver, and spleen, whose disruption results in jaundice and easy bruising. Neurologic manifestations are often subtle, with the delay in motor milestones and hypotonia occurring in the early infantile period, while gait problems, clumsiness, and cataplexy begin presenting in the late infantile-onset cases [1]. Other psychiatric problems, ataxia, dementia, and dystonia, are typical of adult-onset cases. Pulmonary symptoms later manifest as respiratory failure, which is a frequent cause of death.

Diagnosis of Niemann-Pick disease requires history, clinical examination, blood tests, and genetic analysis. At the same time, bone marrow aspiration also aids in diagnosis by demonstrating certain histologic features like the accumulation of foam cells in the marrow. Although genetic analysis is the gold-standard test to confirm the diagnosis of Niemann-Pick disease, developing countries like Pakistan still struggle to provide the facilities needed for such tests to be accessible to the public. Therefore, in this case, genetic testing was not performed since the facilities required for it are not yet available. Treatment of NPD is usually supportive since there is, unfortunately, no cure at the moment. Miglustat is given to slow the neurological symptoms in type C along with supportive therapy, but even in type B and C, which carry relatively better prognosis when compared to type A, patients usually only survive until their teens [1]. Although there is no cure, several studies are ongoing to procure a positive outcome for the patients who suffer from this disease, especially type C. In fact, cyclodextrins (CDs) are the most relevant of the proposed treatments. These nanocarriers are capable of forming complexes with cholesterol which in turn slows the progress of the disease. Many researchers are studying the various forms of these compounds in their use to address this pathology [11].

## Conclusions

Niemann-Pick disease is rare in South-East Asian countries, being more prevalent in the West among Ashkenazi Jews, especially type A Niemann-Pick disease. Additionally, the disease exhibits variable clinical presentation, making it even more difficult to diagnose. There is a high index of suspicion when the presentation includes hepatosplenomegaly, delayed milestones, coarse facial features, and seizures. A definite diagnosis can only be achieved through gene sequencing and sphingomyelinase levels. Unfortunately, in developing countries that lack sufficient medical infrastructure, genetic sequencing studies are most often inaccessible. In addition, bone marrow biopsies are only available in a few facilities within the region. There is no definite treatment for this disease, and mainly supportive treatment and counseling are advised. This case report would add to the sparse literature reporting this disease. Bone marrow trephine helped in the prompt diagnosis and management of this patient.
